# Integrative multi-omics analysis identifies TGFA as a novel glioma susceptibility gene and therapeutic target

**DOI:** 10.3389/fneur.2025.1656490

**Published:** 2025-11-05

**Authors:** Ruiqi Jiang, Shaohua Tu, Nan Ji, Gang Liu, Kefu Yu, Zhigang Zhao

**Affiliations:** ^1^School of Pharmaceutical Sciences, Capital Medical University, Fengtai, Beijing, China; ^2^Department of Pharmacy, Beijing Tiantan Hospital, Capital Medical University, Fengtai, Beijing, China; ^3^Department of Neurosurgery, Beijing Tiantan Hospital, Capital Medical University, Fengtai, Beijing, China; ^4^Department of Infection Control, the Fourth Affiliated Hospital of School of Medicine, and International School of Medicine, International Institutes of Medicine, Zhejiang University, Yiwu, China

**Keywords:** glioma, TGFA, multi-omics analysis, transcriptome-wide association study, drug repurposing

## Abstract

**Background:**

Gliomas are among the most aggressive brain tumors, with high mortality and limited treatments. Despite genetic advances, their molecular mechanisms remain unclear, hindering diagnostic biomarkers and targeted therapies. This study investigates novel glioma susceptibility genes using integrative multi-omics.

**Methods:**

Cross-tissue transcriptome-wide association analyses integrated glioma GWAS data with eQTLs from 49 GTEx v8 tissues, utilizing UTMOST (cross-tissue), FUSION (single-tissue), and MAGMA (gene-level). Prioritized genes underwent Mendelian randomization, Bayesian colocalization, and phenome-wide association. TGFA expression was assessed in glioma samples via public genomic repositories and immunohistochemistry. Drug repurposing employed Comparative Toxicogenomics Database (CTD) and CB-Dock2 for molecular docking.

**Results:**

Five candidate genes were identified (SLC16A8, TGFA, PLA2G6, MAFF, TMEM184B), with Transforming Growth Factor Alpha (TGFA) as the strongest candidate. TGFA showed significant glioma associations across brain tissues and causal relationships via Mendelian randomization (OR: 1.27–1.39), supported by Bayesian colocalization. Elevated TGFA expression occurred in WHO grade 2/3 gliomas and 1p/19q co-deleted tumors, validated by immunohistochemistry. Drug repurposing identified 40 FDA-approved TGFA-targeting drugs; irinotecan exhibited the highest binding affinity (−62.0 kcal/mol) in docking studies.

**Discussion:**

TGFA is a novel glioma susceptibility gene with subtype-specific expression. Its therapeutic targeting offers opportunities for precision therapy, potentially advancing glioma clinical management.

## 1 Introduction

Gliomas constitute a substantial public health burden, representing over 20% of primary brain and central nervous system (CNS) tumors and 80.9% of adult malignant CNS neoplasms, with incidence rates of 30–80 cases per million annually (1, 2). Despite therapeutic advancements, they remain highly aggressive malignancies marked by poor survival and limited treatment efficacy (3). The 2021 WHO classification of CNS tumors categorizes gliomas into four grades: grades 1–2 represent low-grade neoplasms, while grades 3–4 are classified as high-grade tumors (4). Glioblastoma (GBM; grade 4), the most lethal subtype, exhibits a 5-year survival rate of <7% (2).

Although significant advances have been made in elucidating the genetic basis of gliomas, the comprehensive molecular mechanisms and key susceptibility genes driving glioma pathogenesis remain incompletely defined (5). This lack of understanding critically hinders progress toward identifying precise diagnostic biomarkers and developing molecularly targeted treatments, underscoring the pressing requirement for multidisciplinary genomic strategies to clarify the intricate genetic landscape of these tumors. Transforming growth factor alpha (TGF-*α*), a protein encoded by the TGFA gene, belongs to the epidermal growth factor (EGF) family and has functional similarities with EGF in mediating biological processes. It has functional similarities with EGF in mediating biological processes (6). Functionally, TGF-*α* is a ligand for epidermal growth factor receptor (EGFR), which belongs to the receptor tyrosine kinase (RTK) family (7). EGFR alterations represent one of the most common molecular hallmarks of gliomas (8), however, the contribution of its upstream ligand TGFA to gliomagenesis has remained largely unexplored. Emerging evidence suggests that TGF-*α*/EGFR signaling plays a pivotal role in tumor cell proliferation, differentiation, and survival, raising the possibility that TGFA itself may represent a novel glioma susceptibility locus and therapeutic target (7, 9–11).

Recent advances in multi-omics research have offered promising insights into glioma genetics and potential therapeutic targets. Howell et al. (12) demonstrated how DNA methylation modulates glioma risk factors, while Zhou (13). employed Mendelian randomization to identify metabolic alterations associated with glioblastoma. Thornton et al. (11) further leveraged multi-omic MR to uncover druggable targets, demonstrating causal influences from 22 molecular characteristics encompassing 18 genes/proteins on glioma susceptibility. Robinson et al. (9) integrated multi-tissue eQTLs with glioma GWAS data to identify five candidate tissues and four genes previously tied to glioma pathogenesis (JAK1, STMN3, PICK1, and EGFR). Additionally, Zhao et al. (14) explored a causal relationship between *β*-receptor blockers targeting ADRB1 and the development of GBM. Despite these valuable contributions, most studies have primarily focused on investigating gene associations in single tissues, with limited validation across multiple brain regions and limited translation toward therapeutic applications.

Transcriptome-wide association study (TWAS) are used to prioritize potential gene candidates and explore gene-trait connections through integrated analysis of GWAS summary statistics and expression quantitative trait loci (eQTL) datasets (15). UTMOST (Unified Test for Molecular Signature), a methodology for cross-tissue TWAS, expands this framework by simultaneously performing gene-level association analyses across diverse tissues, thereby improving the detection of tissue-specific and shared genetic effects (16). Unlike single-tissue methods, UTMOST employs a “group-lasso penalty” that improves imputation models by identifying shared eQTL effects while preserving tissue-specific variations. This cross-tissue methodology has effectively uncovered candidate genes linked to susceptibility to multiple pathologies, including rheumatoid arthritis (17), essential hypertension (18), and carcinoma of the lung (19).

Our work introduces an integrative multi-omics framework designed to address key methodological limitations in contemporary glioma studies. Through integration of glioma GWAS datasets with GTEx v8 eQTL profiles from 49 tissues, we implement UTMOST for cross-tissue transcriptome-wide association analyses, FUSION (Functional Summary-based Imputation) for tissue-specific evaluations, and MAGMA for gene-level association testing. To validate the robustness of our findings, we employ Mendelian randomization (MR), Bayesian colocalization, and phenome-wide association studies (PheWAS). Furthermore, we extend beyond genetic association to explore therapeutic potential through drug repurposing and molecular docking analyses using the Comparative Toxicogenomics Database (CTD), ChEMBL, and CB-Dock2. This integrated approach not only aims to identify novel glioma susceptibility genes but also to evaluate their potential as therapeutic targets, thereby advancing both our understanding of glioma biology and potential treatment strategies.

## 2 Materials and methods

**Figure 1:**
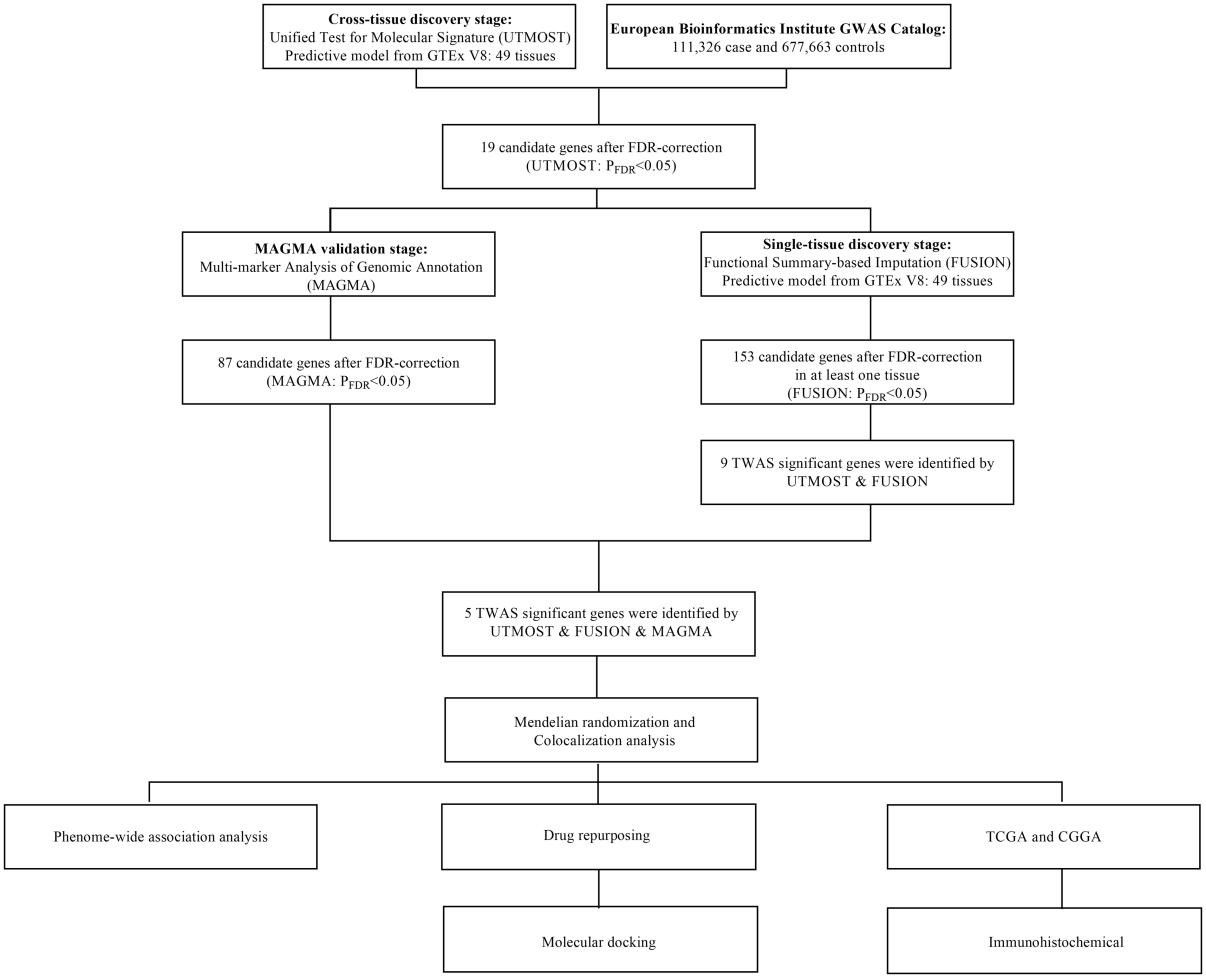
The flowchart of this study. GWAS, genome-wide association; GTEx, Genotype-Tissues Expression Project; TWAS, transcriptome-wide association studies; UTMOST, unified test for molecular signatures; FUSION, functional summary-based imputation; MAGMA, multi-marker Analysis of GenoMic Annotation; TCGA, The Cancer Genome Atlas; CGGA, Chinese Glioma Genome Atlas.

Figure 1 illustrates the methodological framework. This approach combined multi-tissue TWAS via UTMOST, tissue-specific evaluations using FUSION, and MAGMA-based gene testing, following established protocols (20).

### 2.1 Data acquisition

Instrumental variables were selected as cis-single nucleotide polymorphism (cis-SNP) significantly associated with plasma protein levels at the genome-wide significance threshold (*p* < 5 × 10^−8^) from the European Genome Phenotype Archive^1^ database. Cis-SNPs were defined as single nucleotide polymorphisms located within 1 Mb of the gene encoding the respective protein. Linkage disequilibrium (LD) was calculated using data from the 1,000 Genomes European reference panel. Within a 1 Mb window, SNPs with LD values (*r*^2^) less than 0.001 were considered independent. Summary statistics for glioma GWAS were derived from a 2017 publication, encompassing 12,496 glioma cases and 18,190 controls (21). The dataset used in this study is accessible through the European Genome Phenotype Archive (see text footnote 1, respectively) under accession number EGAD00010001657. A total of 26 glioma tissue specimens were acquired from an equal number of patients who underwent neurosurgical procedures at Beijing Tiantan Hospital. None of the individuals participating in the study had received chemotherapy or radiotherapy before sample collection. Approval for the research protocol was provided by the institutional review board of Beijing Tiantan Hospital (approval number: KY2024-346-03), which exempted written informed consent due to the retrospective nature of the research. All protocols adhered to the Declaration of Helsinki and applicable national guidelines.

### 2.2 eQTL data source

The GTEx V8 dataset (22) offers an extensive collection of RNA expression information across 49 distinct tissue types, obtained from 838 deceased donors.^2^ All data are publicly accessible and can be freely retrieved from the GTEx portal for research use. The number of samples varies considerably between tissue types, with renal cortex having the smallest sample size at 73 cases and skeletal muscle representing the largest with 706 cases.

### 2.3 TWAS analyses across tissues

To investigate gene-phenotype associations at a systemic level, we applied UTMOST methodology,^3^ which enables transcriptome-wide analysis across multiple tissues. This approach enhances the capability to identify genes in tissues with stronger signals for heritable traits and improves attribution precision (16, 17). Subsequently, we utilized the generalized Berk-Jones (GBJ) test to incorporate gene-trait associations using the covariance structure derived from tissue-specific summary statistics (16, 23). Statistical significance was determined using false discovery rate (FDR) adjustment, with results considered significant at FDR < 0.05.

### 2.4 TWAS analyses in individual tissues

We employed the FUSION framework^4^ to conduct TWAS by combining glioma GWAS summary statistics with eQTL profiles from 49 tissues in the GTEx V8 dataset (24). To model gene-SNP relationships, we calculated linkage disequilibrium (LD) using individuals of European ancestry from the 1,000 Genomes Project as a reference. FUSION incorporates multiple expression prediction models—BLUP, BSLMM, LASSO, elastic net, and top1—to evaluate SNP effects on gene expression. The model demonstrating optimal predictive performance was selected to generate gene-specific expression weights (25). These weights were subsequently integrated with glioma GWAS Z scores to perform TWAS analysis of glioma susceptibility. Only genes meeting two criteria were retained for final interpretation: (1) FDR < 0.05 in the cross-tissue TWAS and (2) FDR < 0.05 in at least one tissue-specific TWAS result.

### 2.5 Gene analysis

Gene-level analyses were performed using MAGMA (v1.08) under default parameters, consolidating SNP-based association data into gene-level scores to quantify phenotype-linked genetic effects per gene (26). For specific parameters and methodological details, see the original MAGMA documentation (27).

### 2.6 MR and bayesian colocalization

We implemented the “TwoSampleMR” package in R to conduct MR analyses (28). In this investigation, cis-eQTL SNPs served as instrumental variables (IVs), with gene expression as the exposure variable and glioma GWAS statistics as the outcome. SNPs achieving genome-wide significance (*p* < 5 × 10^−8^) were filtered, and LD clustering (*r*^2^ < 0.001) was applied to ensure variant independence (29). For loci containing only one independent IV, causal effects were estimated using the Wald ratio, with *p* < 0.05 as the significance threshold. Bayesian colocalization was conducted using the R package “coloc” (19, 30) to determine whether GWAS and eQTL associations at a specific locus likely share the same causal variant. The posterior probabilities (PP) of five causal models were calculated (19, 30), among which hypothesis 4 (PP. H4) had a PP exceeding 0.75, which was considered as strong evidence supporting the existence of a common genetic basis between the two signals (18, 19).

### 2.7 PheWAS

We used the AstraZeneca PheWAS portal to perform phenotype-wide association studies (PheWAS)^5^ to assess potential pleiotropic effects and unintended consequences of candidate drug targets (31). This approach uses the UK Biobank cohort data (31) to assess the association between rare protein-coding variants within genes and 18,780 phenotypic characteristics. To minimize false-positive findings, we implemented multiple testing correction and established the genome-wide significance threshold at 2 × 10^−8^, consistent with the portal’s default parameters.

### 2.8 Drug repurposing

To obtain insights into the drug compounds targeting proteins identified in this study, we utilized the CTD^6^ (32) (as of 16/3/2025). This database offers manually curated information on 50 million toxicogenomic relationships. Our analysis focused primarily on compounds with experimentally validated interactions with protein targets. To ensure translational relevance, only FDA-approved drugs were considered for subsequent docking analysis. Furthermore, we identified drugs aimed at these two proteins that are currently undergoing clinical trials using the ChEMBL (33) database (as of 16/3/2025).

### 2.9 Molecular docking

Molecular docking provides a computational framework for evaluating the affinity and interaction between candidate ligands and corresponding molecular targets, thereby facilitating the screening of promising therapeutic drugs for further experimental evaluation and drug development improvements. This study used the web-based tool CB-Dock2^7^ (34) developed by Cao Yang’s laboratory for molecular docking simulations. The researchers used compounds from the Comparative Toxicogenomics Database (CTD) to screen proteins encoded by genes associated with the pathogenesis of glioma (35).

For each ligand, CB-Dock2 automatically identified the top five binding cavities ranked by cavity volume. Docking was performed in all five cavities, and the vina scoring function was used to calculate binding affinity values (kcal/mol). Docking outputs included cavity center coordinates, cavity size, docking poses, and binding energies. The best-scoring conformation (lowest binding energy) was used for downstream analysis of protein–ligand interactions.

The three-dimensional structures of four bioactive molecules—estradiol (PubChem CID: 5757), gefitinib (CID: 123631), irinotecan (CID: 60838), and bromocriptine (CID: 31101)—were obtained in sdf format from the PubChem database.^8^ The crystal structure of transforming growth factor *α* (TGF-α) (PDB ID 1YUF) was acquired in pdb format from the Protein Data Bank.^9^

### 2.10 Gene expression analysis using public genomic repositories

Transcriptomic information and associated clinical data from glioma specimens were extracted from two major genomic databases: the Chinese Glioma Genome Atlas^10^ and The Cancer Genome Atlas.^11^ After excluding entries with incomplete clinical annotations or insufficient follow-up, a gene expression matrix was generated for prognostic analysis. The relationship between TGFA expression level and glioma histological grade was evaluated using R software (version 4.3.0).

### 2.11 Immunohistochemical staining protocol

Formalin-fixed, paraffin-embedded glioma tissue sections were initially heated at 60 °C for 90 min. Deparaffinization was performed by immersing the sections in xylene three times, each for 5 min, followed by sequential rehydration through descending ethanol concentrations (100, 95, and 75%). After three washes with PBS (5 min each), endogenous peroxidase activity was blocked with 3% hydrogen peroxide solution for 10 min at room temperature. Heat-induced epitope retrieval involved immersing slides in antigen retrieval buffer at 95 °C for 10 min, then allowing them to cool gradually to ambient temperature. Permeabilization was carried out with 0.1% Triton X-100 in PBS for 5 min, and nonspecific binding was blocked using 1% bovine serum albumin (BSA) for 30 min. Slides were incubated overnight at 4 °C with an anti-TGF-*α* primary antibody (Immunoway, YT4626) diluted 1:100 in blocking buffer. The following day, after three additional PBS washes (10 min each), sections were treated with an HRP-conjugated goat anti-rabbit secondary antibody for 60 min. Signal detection was achieved using 3,3′-diaminobenzidine (DAB) substrate, and the reaction was halted with tap water. Hematoxylin was applied for nuclear counterstaining (1 min), followed by differentiation in 1% acid-alcohol. Slides were mounted using neutral resin and digitized via the Leica Aperio AT2 scanning system. Staining intensity was quantified using ImageJ, and statistical analysis was conducted using GraphPad Prism version 8 (GraphPad Software, San Diego, CA, United States), with statistical significance defined as *p* < 0.05.

## 3 Results

### 3.1 TWAS findings from cross-tissue and single-tissue analyses

TWAS studies conducted through both cross-tissue and single-tissue analytical frameworks identified distinct sets of genes associated with glioma susceptibility. The cross-tissue analysis revealed 218 genes with nominal significance (*p* < 0.05; Supplementary Table S1), of which 18 remained significant after FDR adjustment (FDR < 0.05; Table 1). Parallel analysis in individual tissues confirmed that 153 genes achieved FDR < 0.05 in at least one tissue type (Supplementary Table S2). A subset of 9 protein-coding genes, POLR2F, SLC16A8, CPSF3, PXDN, TGFA, PLA2G6, MAFF, TMEM184B, and CSNK1E, met statistical criteria in both analysis models, indicating that they are reliable and high-confidence candidate genes for glioma susceptibility.

**Table 1:** The significant genes for glioma risk in cross-tissue UTMOST analysis.

Gene symbole	CHR	Ensemeble ID	Location (hg38)	Test score	*p* value	FDR
EIF3L	22	ENSG00000100129	37,848,868–37,889,407	8.57	1.19E-04	3.38E-02
POLR2F	22	ENSG00000100142	37,952,607–38,041,915	27.95	2.43E-13	8.98E-10
SLC16A8	22	ENSG00000100156	38,078,134–38,084,184	9.70	3.78E-05	1.47E-02
TOMM22	22	ENSG00000100216	38,681,957–38,685,421	13.74	4.56E-07	4.22E-04
RASD2	22	ENSG00000100302	35,540,831–35,553,999	8.61	1.94E-04	3.91E-02
NMU	4	ENSG00000109255	55,595,229–55,636,698	8.46	2.01E-04	3.91E-02
CPSF3	2	ENSG00000119203	9,423,651–9,473,101	7.89	1.93E-04	3.91E-02
PXDN	2	ENSG00000130508	1,631,887–1,744,852	10.01	2.78E-05	1.47E-02
TGFA	2	ENSG00000163235	70,447,284–70,554,193	9.96	3.42E-05	1.47E-02
KCNJ4	22	ENSG00000168135	38,426,327–38,455,199	9.44	6.28E-05	2.11E-02
PCBP1	2	ENSG00000169564	70,087,477–70,089,203	9.30	1.45E-04	3.84E-02
KLF11	2	ENSG00000172059	10,042,849–10,054,836	10.81	1.79E-05	1.11E-02
PCBP1-AS1	2	ENSG00000179818	69,960,104–70,103,220	7.81	1.81E-04	3.91E-02
PLA2G6	22	ENSG00000184381	38,111,495–38,214,778	7.56	9.31E-05	2.87E-02
MAFF	22	ENSG00000185022	38,200,767–38,216,507	9.81	3.98E-05	1.47E-02
H1-0	22	ENSG00000189060	37,805,229–37,807,432	8.79	1.69E-04	3.91E-02
TMEM184B	22	ENSG00000198792	38,219,291–38,273,010	14.10	1.73E-07	3.21E-04
CSNK1E	22	ENSG00000213923	38,290,691–38,318,084	11.62	3.44E-06	2.55E-03

### 3.2 MAGMA gene-based analysis

**Figure 2:**
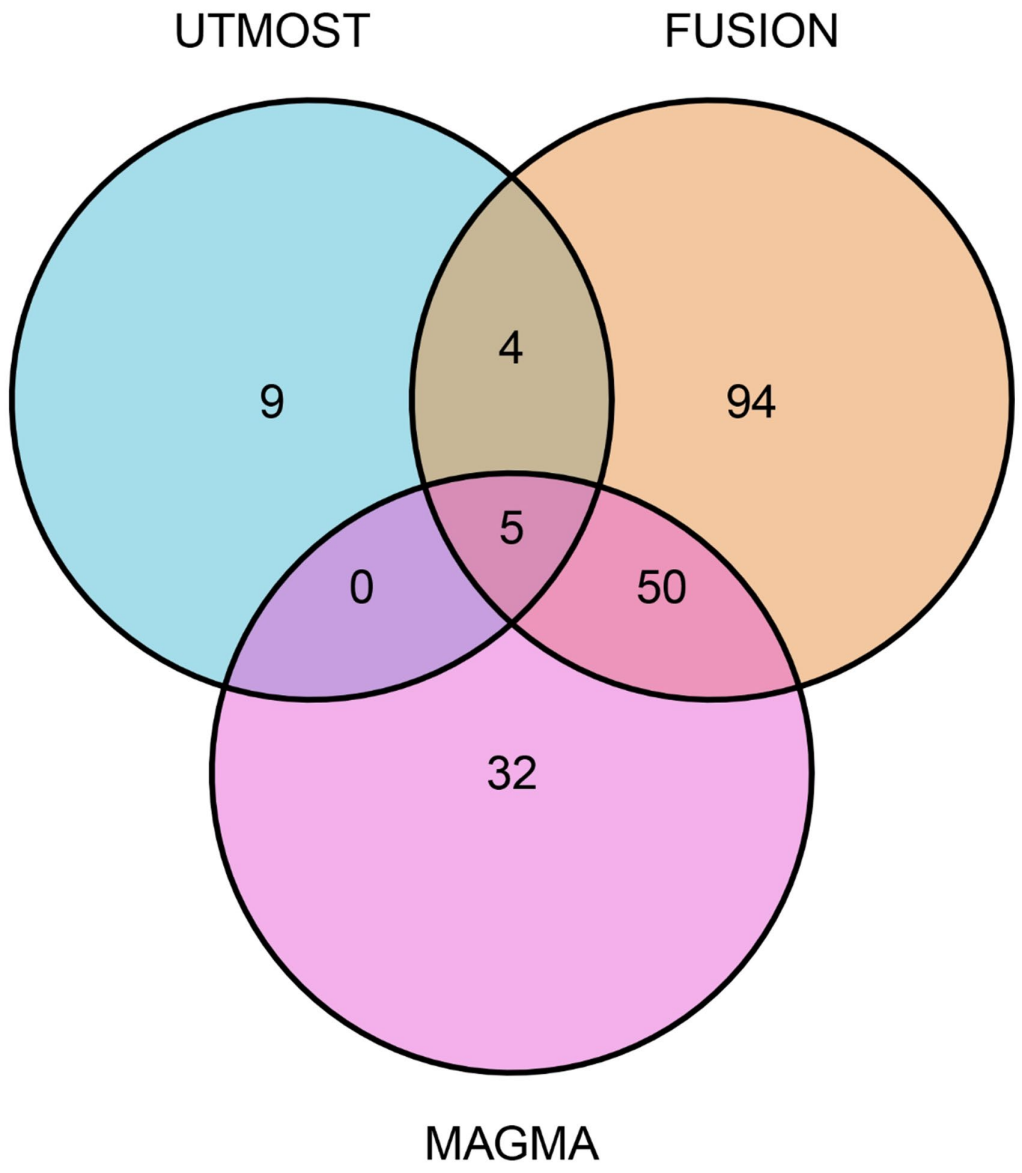
Venn diagram. MAGMA identified 87 significant genes associated with glioma, FUSION identified 153, and UTMOST cross-tissue analysis identified 18.

Using MAGMA for gene-level association testing, we identified 87 genes significantly linked to glioma susceptibility after applying FDR correction (FDR < 0.05; Supplementary Table S3). We combined the results from the UTMOST cross-tissue TWAS analysis with prioritized genes from both FUSION and MAGMA analyses to strengthen the reliability of candidate gene screening. This integrated approach highlighted five strong candidate genes with consistent signals in different methods: SLC16A8, TGFA, PLA2G6, MAFF, and TMEM184B (Figure 2).

### 3.3 MR and colocalization results

The TGFA gene, located on chromosome 2, demonstrated significant associations with glioma across several brain regions—including the caudate basal ganglia, cortex, and hypothalamus—based on FUSION analysis results. MR supported a potential causal effect of TGFA on glioma risk, yielding statistically significant associations (*p* < 0.05). The estimated odds ratios (ORs) and their 95% confidence intervals (CIs) for the corresponding brain regions were 1.39 (1.18–1.64), 1.27 (1.12–1.43), and 1.28 (1.13–1.45), respectively (Supplementary Table S4). Bayesian colocalization reinforced this link, showing high posterior probabilities (PP. H4 = 0.86–0.93) for shared causal variants in all three tissues (Supplementary Table S5). The SNP rs7561547 emerged as the primary colocalized variant for glioma in these regions (Supplementary Figures S1, S2).

### 3.4 PheWAS

To assess therapeutic benefits or unintended effects of TGFA as a glioma drug target and evaluate off-target pleiotropy beyond MR–Egger intercept findings, we performed gene-level PheWAS using 17,361 binary and 1,419 quantitative traits from the AstraZeneca PheWAS Portal (31). This analysis tested associations between genetically predicted TGFA protein levels and diseases/traits. As summarized in Supplementary Table S6 and Supplementary Figure S3, TGFA showed no significant associations (genomic-wide significance threshold: *p* < 2 × 10^−8^), suggesting minimal off-target pleiotropic effects and supporting the specificity of TGFA as a therapeutic target.

### 3.5 Drug repurposing

The CTD database (see text footnote 6, respectively) was queried for TGFA drug targets, revealing 40 FDA-approved drugs with potential for treating glioma among 242 interacting chemicals (Supplementary Table S7). Additionally, a search of the ChEMBL database revealed a drug targeting TGFA, revealing 154 FDA-approved drugs with potential for treating glioma among 464 drugs (Supplementary Table S8). Only bromocriptine was in both two databases.

### 3.6 Molecular docking

Molecular docking studies focused on TGFA, the primary therapeutic target, were conducted with six pharmacological agents using the CB-Dock2 platform. Binding site analyses and interaction profiling for four lead compounds with their respective protein targets revealed energy values for each molecular complex. For each ligand, the top five cavities predicted by CB-Dock2 were explored, and docking was evaluated using the vina scoring function. The results demonstrated that all ligands achieved stable binding conformations with negative docking scores, reflecting favorable protein–ligand interactions. Supplementary Table S9 and Supplementary Figure S4 illustrate the ligand-binding conformations and spatial orientations for the top three drugs by interaction frequency (estradiol, gefitinib, irinotecan) and bromocriptine when docked with TGFA. Supplementary Tables S9-1–S9-4 detail the top five binding cavities, prioritized by volumetric parameters and energy metrics. All evaluated compounds demonstrated stable hydrogen bonding networks and pronounced electrostatic complementarity with their targets. Notably, the TGFA-irinotecan complex displayed optimal binding affinity (−62.0 kcal/mol), reflecting superior molecular stability among the tested pairs.

### 3.7 Gene expression differences in glioma subtypes

**Figure 3:**
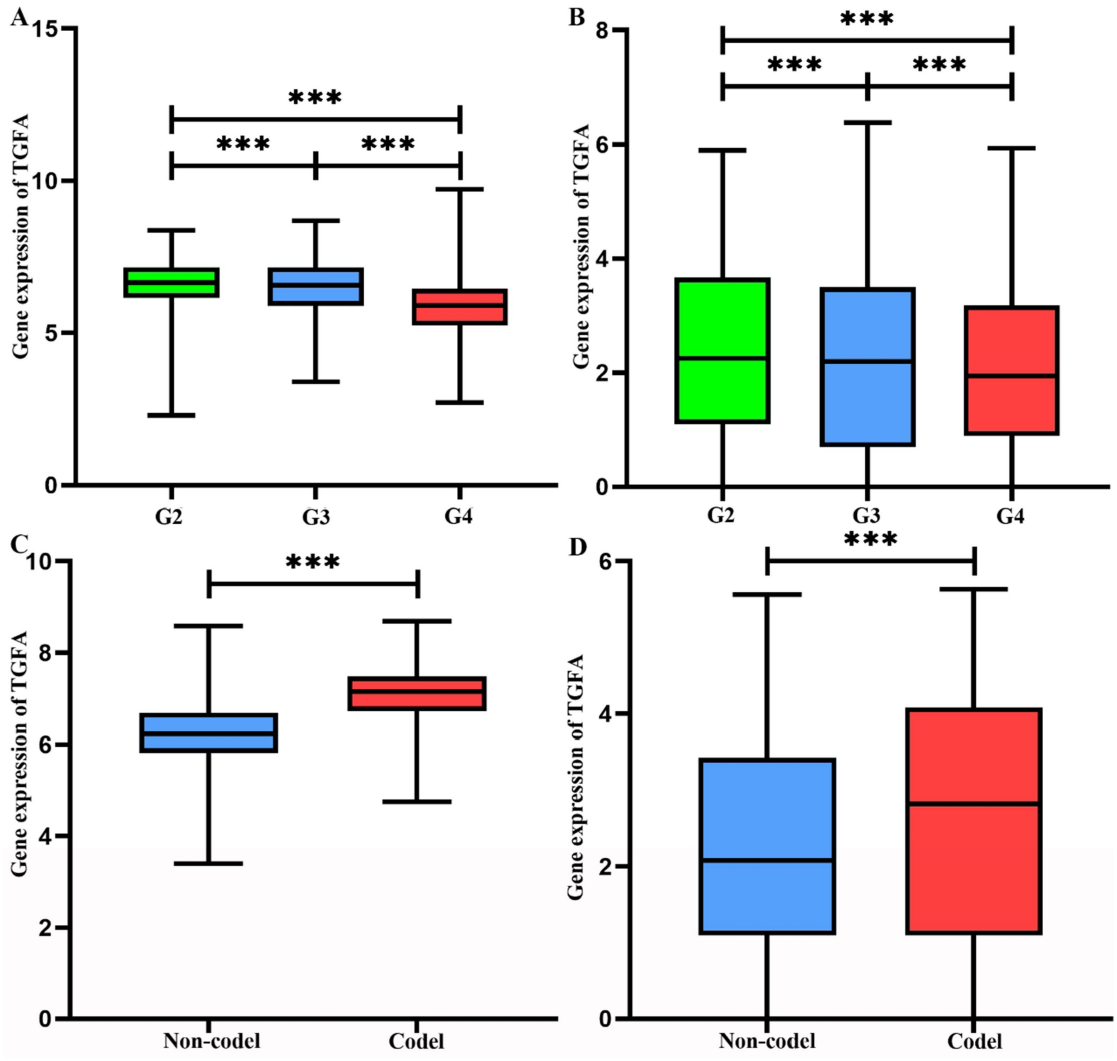
Analysis of TGFA gene expression and genetic alterations in TCGA and CGGA. **(A)** Gene expression levels of TGFA across distinct grades in TCGA. Statistical significance was determined by ANOVA (*p* < 0.001). **(B)** Gene expression levels of TGFA across distinct grades in CGGA. Statistical significance was determined by ANOVA (*p* < 0.001). **(C)** Association between TGFA expression and 1p/19q chromosomal deletion status in TCGA (Non-deletion vs. Co-deletion). Statistical significance was determined by ANOVA (*p* < 0.001). **(D)** Association between TGFA expression and 1p/19q chromosomal deletion status in CGGA (Non-deletion vs. Co-deletion). Statistical significance was determined by ANOVA (*p* < 0.001).

In both the TCGA and CGGA cohorts, WHO grade II and III gliomas had significantly higher TGFA expression levels than grade IV tumors (*p* < 0.0001; Figures 3A,B). Similarly, tumors harboring 1p/19q co-deletion demonstrated significantly upregulated TGFA expression compared to non-codeleted gliomas in these datasets (*p* < 0.0001; Figures 3C,D). These observations imply a possible link between TGFA transcriptional activity and distinct molecular-pathological glioma subtypes.

### 3.8 Clinical glioma samples validate gene signature

**Figure 4:**
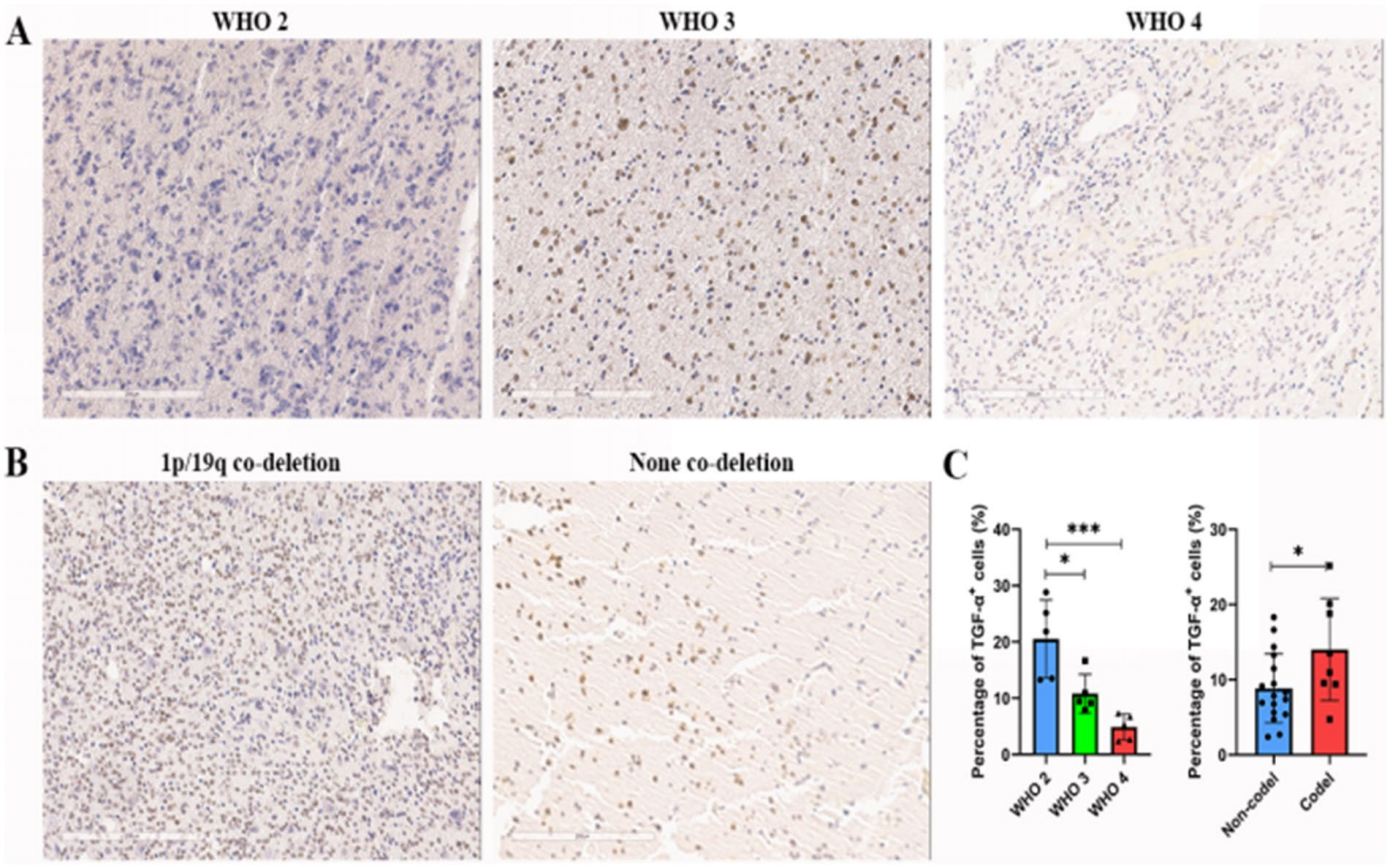
Association between TGFA immunoexpression and clinicopathological features in glioma cases. **(A)** Representative images of IHC staining of TGFA index in glioma samples across distinct grades. **(B)** Representative images of IHC staining of TGFA index in glioma samples across 1p/19q chromosomal deletion status. **(C)** Results of the percentage of TGF-α+ cells of TGFA in distinct grades and 1p/19q chromosomal deletion status. *p < 0.05; **p < 0.01; ***p < 0.001.

Prognostic evaluation of glioma patients was performed via IHC staining of TGFA in surgically resected tissues. TGFA immunoreactivity showed heterogeneous expression patterns but enabled clear differentiation between high- and low-expression subgroups. Notably, TGFA levels were markedly elevated in WHO grade 2 gliomas compared to grades 3–4 (Figure 4A) and in tumors with 1p/19q co-deletion versus non-codeleted cases (Figure 4B), aligning with TCGA and CGGA database findings (Figure 4C). Clinicopathological and molecular correlates are detailed in Supplementary Table S10.

## 4 Discussion

This study used a multi-omics research strategy to elucidate the genetic architecture of glioma susceptibility by coordinating glioma GWAS data with GTEx V8 expression quantitative trait loci. A tiered analytical pipeline—incorporating cross-tissue transcriptome-wide association via UTMOST, tissue-specific TWAS with FUSION, and MAGMA-based gene prioritization—uncovered Transforming Growth Factor Alpha (TGFA) as a novel glioma risk locus. Causal inference analyses (Mendelian randomization and Bayesian colocalization) reinforced TGFA’s role, demonstrating consistent risk effects (OR: 1.27–1.39) and identifying rs7561547 as a shared causal variant (posterior probability: 0.86–0.93) across cerebral tissues. TGFA expression patterns mirrored genetic associations, showing marked upregulation in WHO grade 2–3 gliomas versus grade 4 and in 1p/19q codeleted tumors across TCGA, CGGA, and institutional cohorts (Supplementary Figure S2). Computational drug repositioning highlighted irinotecan as a high-affinity TGFA binder (−62.0 kcal/mol), supported by molecular docking. These results collectively nominate TGFA as a tractable therapeutic target and mechanistic hub in glioma pathogenesis, bridging genetic epidemiology to translational pharmacology.

Multi-omics association studies are increasingly used to investigate glioma pathobiology and identify potential drug repurposing opportunities. For instance, Zhou (13) reported 69 plasma metabolites linked to glioblastoma development. In another study, Robinson et al. combined eQTL data from multiple tissues with a glioma GWAS dataset and identified five glioma-associated brain regions (cerebellum, basal nuclei accumbens, cerebral cortex, caudate basal ganglia, and putamen) along with four associated genes (STMN3, JAK1, EGFR, and PICK1) (9). Utilizing brain multi-omic analyses, Thornton et al. revealed causal relationships between 22 molecular features spanning 18 genes or proteins and glioma susceptibility (11). Similarly, Zhao et al. investigated initial indications of a connection between *β*-receptor blockers targeting ADRB1 and glioblastoma progression by integrating eQTL colocalization with single-cell RNA-seq data (14). Nevertheless, cross-tissue analytical results require further validation, as existing research has largely concentrated on single-tissue gene associations with glioma.

Robinson et al. (9), Thornton et al. (11), and our research reinforce the critical role of brain-tissue specificity, though their objectives diverge. Thornton et al. (11) applied a multi-omics MR approach to identify new therapeutic targets for gliomas and demonstrated the causal role of molecular features. Similarly, Robinson et al. (9) adopted MR but prioritized transcriptome-wide analyses to uncover tissue-dependent genes linked to glioma risk, revealing three previously uncharacterized susceptibility loci and underscoring tissue-specific mechanisms. Unlike these studies, our work combined diverse omics methodologies—such as TWAS, UTMOST, MAGMA, FUSION, and Bayesian colocalization—to map glioma risk genes, with a focus on TGFA as a novel risk factor in the caudate basal ganglia, cortex, and hypothalamus. We further explored TGFA’s therapeutic utility via drug repurposing and molecular docking. A key strength of our approach lies in its methodological breadth, incorporating PheWAS and docking simulations to connect genetic insights to clinical translation. Additionally, we identified FDA-approved drugs targeting TGFA, bolstering translational relevance. By merging multi-layered validation with drug discovery components, our framework offers a more holistic and clinically actionable investigation.

This study’s core analytical framework centered on a cross-tissue TWAS framework, leveraging UTMOST’s integrative model. Unlike conventional single-tissue TWAS, this methodology integrates gene expression data across tissues to bolster statistical power for detecting trait-associated genes, enabling a more comprehensive elucidation of gene-trait relationships and improving the detection of associations that may not be detected in single-tissue analyses (16). In recent years, several studies have adopted multi-tiered analytical pipelines incorporating cross-tissue TWAS screening, single-tissue TWAS, gene-level validation, and colocalization techniques and Mendelian randomization (MR) to map susceptibility genes across diverse pathologies (17, 20, 36). Through cross-tissue TWAS coupled with rigorous validation, we identified TGFA as a previously unreported glioma risk locus.

Recent molecular advances have substantially enriched our understanding of gliomagenesis. For example, large-scale integrative studies have revealed new epigenetic and metabolic drivers of glioma. Howell et al. (12) demonstrated how DNA methylation mediates the effects of established risk factors on glioma incidence, underscoring the role of epigenetic regulation in tumor initiation. Glioma stem-like cells sustained by chromatin regulators such as BRD proteins and PRC2 components, together with arginine and lysine methylation dynamics mediated by PRMTs and KDMs, have emerged as key determinants of self-renewal, DNA repair, and therapy resistance, opening new therapeutic opportunities (37). Moreover, oncogenic signaling through EGFR and PI3K/AKT converges with metabolic rewiring and post-translational modifications, including PKM2 O-GlcNAcylation and PTEN succination, while spatially resolved profiling has emphasized tumor heterogeneity and the roles of neuronal interactions and lipid-laden macrophages in immune evasion (37). These fundamental works unravel the complexities of glioma development and present new options for therapeutic intervention. Our discovery of TGFA as a glioma susceptibility gene adds to the growing body of research by connecting cross-tissue transcriptome connections to functional validation and therapeutic repurposing, thereby expanding the molecular framework of gliomagenesis for translational application.

It is important to acknowledge that TGFA has previously been implicated in gliomas, largely through its role as a ligand of EGFR. Early experimental work demonstrated that the TGF-*α*/EGFR autocrine loop promotes glioma cell proliferation and survival (7), and subsequent studies confirmed EGFR–TGF-*α* signaling as a hallmark pathway in human gliomas arget (10). Animal models have further shown that TGF-α overexpression can drive glioma-like phenotypes (6). However, these investigations were primarily mechanistic or limited to small-scale experimental systems. By contrast, our study is the first to integrate large-scale GWAS, multi-tissue eQTL datasets, and cross-tissue transcriptomic analyses to establish TGFA as a glioma susceptibility gene in human populations. Moreover, we extend this genetic association to translational relevance by demonstrating its potential as a therapeutic target through drug repurposing and molecular docking. Thus, our findings advance prior knowledge by moving from experimental implication of TGFA in gliomagenesis to population-level genetic validation and actionable therapeutic insight.

Estrogen and its receptors show considerable therapeutic potential in glioblastoma treatment due to their ability to modulate multiple biological pathways, cross the blood–brain barrier (BBB), and regulate transcription, making them viable therapeutic candidates (38). For example, Lee et al. demonstrated that 17β-estradiol and tamoxifen upregulated glutamate transporter-1 expression in astrocytes through TGF-*α*-mediated signaling, identifying a critical target for neurological therapy development (39). Gefitinib, a tyrosine kinase inhibitor (TKI) targeting EGFR (40), has shown sensitivity strongly associated with the EGFR ligand TGF-*α* (41). Irinotecan, a topoisomerase I inhibitor and camptothecin analog capable of crossing the BBB, had previously been evaluated in glioma treatment, but with limited clinical success when administered as a conventional topoisomerase I inhibitor (42). Our findings, however, suggest a distinct translational angle. Molecular docking revealed that irinotecan exhibits a strong binding affinity to TGFA, raising the possibility that its activity may extend beyond cytotoxicity to modulation of the TGFA–EGFR signaling axis, a pathway critically implicated in gliomagenesis (43). This mechanistic insight provides a rationale for reconsidering irinotecan not as an empirical chemotherapeutic, but rather as a biomarker-guided therapeutic candidate in TGFA-driven glioma subtypes. More broadly, our results exemplify how integrating genetic epidemiology with drug repurposing can uncover novel applications for existing FDA-approved agents, thereby accelerating the translation of molecular discoveries into targeted therapeutic strategies. Similarly, bromocriptine’s role in glioma therapy remains underexplored, though studies report sustained TGF-α mRNA upregulation during active pituitary tumor growth. Intriguingly, bromocriptine-induced activation of dopamine D2 receptors suppresses TGF-α mRNA expression prior to tumor shrinkage, suggesting its therapeutic promise for glioma (44). Further translational research—spanning both *in vitro* systems and *in vivo* models—remains essential to validate bromocriptine’s therapeutic efficacy and elucidate its mechanistic foundations.

While this study provides novel insights, several limitations warrant acknowledgment. (1) Our analyses relied on GWAS and eQTL datasets derived exclusively from European cohorts, potentially restricting the generalizability of results to populations of non-European ancestries. Validation in increasingly diverse cohorts is critical to ensuring the relevance of these findings across communities. (2) Although the study revealed substantial insights through computational and statistical analysis, there was no direct experimental validation of the relationships between TGFA and glioma. Additional biological investigations are required to validate these findings and investigate their functional consequences. (3) Although the study discovered possible TGFA genes associated with glioma, it did not completely understand the molecular mechanisms behind their roles in tumor progression. Further mechanistic research, including functional assays, is required to establish causal links.

## 5 Conclusion

To identify glioma susceptibility genes, this study employed a comprehensive multi-omics technique that comprised cross- and single-tissue TWAS, MAGMA, MR, and Bayesian colocalization. Our findings consistently identified TGFA as a distinct risk gene, with high associations in the caudate basal ganglia, brain, and hypothalamus. Mendelian randomization revealed a connection between higher TGFA expression and increased glioma risk, whereas colocalization analysis revealed common causal variants. Furthermore, phenome-wide association studies indicated minimal pleiotropic effects, underlining the importance of TGFA participation. Bromocriptine was identified as a potential treatment approach through medication repurposing and molecular docking studies due to its favorable binding energy to TGFA. Despite the study’s limitations, which include European-centric datasets and a lack of experimental confirmation, the findings lay a solid platform for future research. These findings will need to be translated into therapeutic applications through further cohort research and functional testing. Overall, our findings provide substantial contributions to glioma research and suggest promising future directions.

## Data Availability

The original contributions presented in the study are included in the article/Supplementary material, further inquiries can be directed to the corresponding authors.

## Footnotes

^1^EGA; http://www.ebi.ac.uk/ega/

^2^ https://ftp.ebi.ac.uk/pub/databases/spot/eQTL/imported/GTEx_V8

^3^ https://github.com/Joker-Jerome/UTMOST?tab=readme-ov-file

^4^ http://gusevlab.org/projects/fusion/

^5^ https://azphewas.com/

^6^ http://ctdbase.org/

^7^https://cadd.labshare.cn/cb-dock2/index.php; access date: March 16, 2025

^8^https://pubchem.ncbi.nlm.nih.gov/; accessed March 16, 2025

^9^https://www.rcsb.org/; accessed March 16, 2025

^10^CGGA; http://www.cgga.org.cn

^11^TCGA; https://www.cancer.gov/tcga

## References

[1] Li T Li J Chen Z Zhang S Li S Wageh S. Glioma diagnosis and therapy: current challenges and nanomaterial-based solutions. J Control Release. (2022) 352:338–70. doi: 10.1016/j.jconrel.2022.09.065, PMID: 36206948

[2] Ostrom QT Cioffi G Waite K Kruchko C Barnholtz-Sloan JS. CBTRUS statistical report: primary brain and other central nervous system Tumors diagnosed in the United States in 2014-2018. Neuro-Oncology. (2021) 23:iii1–iii105. doi: 10.1093/neuonc/noab200, PMID: 34608945 PMC8491279

[3] Ostrom QT Bauchet L Davis FG Deltour I Fisher JL Langer CE. The epidemiology of glioma in adults: a "state of the science" review. Neuro-Oncology. (2014) 16:896–913. doi: 10.1093/neuonc/nou087, PMID: 24842956 PMC4057143

[4] Miller KD Ostrom QT Kruchko C Patil N Tihan T Cioffi G. Brain and other central nervous system tumor statistics, 2021. CA Cancer J Clin. (2021) 71:381–406. doi: 10.3322/caac.21693, PMID: 34427324

[5] Khiabani NA Doustvandi MA Story D Nobari SA Hajizadeh M Petersen R. Glioblastoma therapy: state of the field and future prospects. Life Sci. (2024) 359:123227. doi: 10.1016/j.lfs.2024.123227, PMID: 39537100

[6] Gangarosa LM Dempsey PJ Damstrup L Barnard JA Coffey RJ. Transforming growth factor-alpha. Baillieres Clin Gastroenterol. (1996) 10:49–63. doi: 10.1016/s0950-3528(96)90039-1, PMID: 8732300

[7] Tang P Steck PA Yung WK. The autocrine loop of TGF-alpha/EGFR and brain tumors. J Neuro-Oncol. (1997) 35:303–14. doi: 10.1023/a:1005824802617, PMID: 9440027

[8] Oprita A Baloi SC Staicu GA Alexandru O Tache DE Danoiu S. Updated insights on EGFR Signaling pathways in glioma. Int J Mol Sci. (2021) 22:587. doi: 10.3390/ijms22020587, PMID: 33435537 PMC7827907

[9] Robinson JW Martin RM Tsavachidis S Howell AE Relton CL Armstrong GN. Transcriptome-wide mendelian randomization study prioritising novel tissue-dependent genes for glioma susceptibility. Sci Rep. (2021) 11:2329. doi: 10.1038/s41598-021-82169-5, PMID: 33504897 PMC7840943

[10] Saadeh FS Mahfouz R Assi HI. EGFR as a clinical marker in glioblastomas and other gliomas. Int J Biol Markers. (2018) 33:22–32. doi: 10.5301/ijbm.5000301, PMID: 28885661

[11] Thornton ZA Andrews LJ Zhao H Zheng J Paternoster L Robinson JW. Brain multi-omic mendelian randomisation to identify novel drug targets for gliomagenesis. Hum Mol Genet. (2025) 34:178–92. doi: 10.1093/hmg/ddae168, PMID: 39565278 PMC11780873

[12] Howell AE Relton C Martin RM Zheng J Kurian KM. Role of DNA methylation in the relationship between glioma risk factors and glioma incidence: a two-step mendelian randomization study. Sci Rep. (2023) 13:6590. doi: 10.1038/s41598-023-33621-1, PMID: 37085538 PMC10121678

[13] Zhou Z Leng H. Deciphering the causal relationship between plasma and cerebrospinal fluid metabolites and glioblastoma multiforme: a mendelian randomization study. Aging (Albany NY). (2024) 16:8306–19. doi: 10.18632/aging.205818, PMID: 38742944 PMC11131984

[14] Zhao S Xie Y Ding X Zheng C Chen J Zhao N. Exploring the causal relationship between antihypertensive drugs and glioblastoma by combining drug target mendelian randomization study, eQTL colocalization, and single-cell RNA sequencing. Environ Toxicol. (2024) 39:3425–33. doi: 10.1002/tox.24210, PMID: 38450887

[15] Gamazon ER Wheeler HE Shah KP Mozaffari SV Aquino-Michaels K. A gene-based association method for mapping traits using reference transcriptome data. Nat Genet. (2015) 47:1091–8. doi: 10.1038/ng.3367, PMID: 26258848 PMC4552594

[16] Hu Y Li M Lu Q Weng H Wang J Zekavat SM. A statistical framework for cross-tissue transcriptome-wide association analysis. Nat Genet. (2019) 51:568–76. doi: 10.1038/s41588-019-0345-7, PMID: 30804563 PMC6788740

[17] Ni J Wang P Yin KJ Yang XK Cen H Sui C. Novel insight into the aetiology of rheumatoid arthritis gained by a cross-tissue transcriptome-wide association study. RMD Open. (2022) 8:e002529. doi: 10.1136/rmdopen-2022-002529, PMID: 37582060 PMC9462377

[18] Huang S Wang J Liu N Li P Wu S Qi L. A cross-tissue transcriptome association study identifies key genes in essential hypertension. Front Genet. (2023) 14:1114174. doi: 10.3389/fgene.2023.1114174, PMID: 36845374 PMC9950398

[19] Zhu M Fan J Zhang C Xu J Yin R Zhang E. A cross-tissue transcriptome-wide association study identifies novel susceptibility genes for lung cancer in Chinese populations. Hum Mol Genet. (2021) 30:1666–76. doi: 10.1093/hmg/ddab119, PMID: 33909040

[20] Gui J Yang X Tan C Wang L Meng L Han Z. A cross-tissue transcriptome-wide association study reveals novel susceptibility genes for migraine. J Headache Pain. (2024) 25:94. doi: 10.1186/s10194-024-01802-6, PMID: 38840241 PMC11151630

[21] Melin BS Barnholtz-Sloan JS Wrensch MR Johansen C Il'yasova D Kinnersley B. Genome-wide association study of glioma subtypes identifies specific differences in genetic susceptibility to glioblastoma and non-glioblastoma tumors. Nat Genet. (2017) 49:789–94. doi: 10.1038/ng.3823, PMID: 28346443 PMC5558246

[22] Ocaranza P Quintanilla ME Tampier L Karahanian E Sapag A Israel Y. Gene therapy reduces ethanol intake in an animal model of alcohol dependence. Alcohol Clin Exp Res. (2008) 32:52–7. doi: 10.1111/j.1530-0277.2007.00553.x, PMID: 18070247

[23] Sun R Hui S Bader GD Lin X Kraft P. Powerful gene set analysis in GWAS with the generalized Berk-Jones statistic. PLoS Genet. (2019) 15:e1007530. doi: 10.1371/journal.pgen.1007530, PMID: 30875371 PMC6436759

[24] Gusev A Ko A Shi H Bhatia G Chung W Penninx BWJH. Integrative approaches for large-scale transcriptome-wide association studies. Nat Genet. (2016) 48:245–52. doi: 10.1038/ng.3506, PMID: 26854917 PMC4767558

[25] Li S-J Shi J-J Mao C-Y Zhang C Xu Y-F Fan Y. Identifying causal genes for migraine by integrating the proteome and transcriptome. J Headache Pain. (2023) 24:111. doi: 10.1186/s10194-023-01649-3, PMID: 37592229 PMC10433568

[26] De Leeuw CA Neale BM Heskes T Posthuma D. The statistical properties of gene-set analysis. Nat Rev Genet. (2016) 17:353–64. doi: 10.1038/nrg.2016.29, PMID: 27070863

[27] De Leeuw CA Mooij JM Heskes T Posthuma D. MAGMA: generalized gene-set analysis of GWAS data. PLoS Comput Biol. (2015) 11:e1004219. doi: 10.1371/journal.pcbi.1004219, PMID: 25885710 PMC4401657

[28] Hemani G Zheng J Elsworth B Wade KH Haberland V Baird D. The MR-base platform supports systematic causal inference across the human phenome. eLife. (2018) 7:408. doi: 10.7554/eLife.34408, PMID: 29846171 PMC5976434

[29] Gui J Meng L Huang D Wang L Yang X Ding R. Identification of novel proteins for sleep apnea by integrating genome-wide association data and human brain proteomes. Sleep Med. (2024) 114:92–9. doi: 10.1016/j.sleep.2023.12.026, PMID: 38160582

[30] Giambartolomei C Vukcevic D Schadt EE Franke L Hingorani AD Wallace C. Bayesian test for colocalisation between pairs of genetic association studies using summary statistics. PLoS Genet. (2014) 10:e1004383. doi: 10.1371/journal.pgen.1004383, PMID: 24830394 PMC4022491

[31] Wang Q Dhindsa RS Carss K Harper AR Nag A Tachmazidou I. Rare variant contribution to human disease in 281,104 UK biobank exomes. Nature. (2021) 597:527–32. doi: 10.1038/s41586-021-03855-y, PMID: 34375979 PMC8458098

[32] Davis AP Wiegers TC Johnson RJ Sciaky D Wiegers J Mattingly CJ. Comparative Toxicogenomics database (CTD): update 2023. Nucleic Acids Res. (2023) 51:D1257–d1262. doi: 10.1093/nar/gkac833, PMID: 36169237 PMC9825590

[33] Gaulton A Bellis LJ Bento AP Chambers J Davies M Hersey A. ChEMBL: a large-scale bioactivity database for drug discovery. Nucleic Acids Res. (2012) 40:D1100–7. doi: 10.1093/nar/gkr777, PMID: 21948594 PMC3245175

[34] Liu Y Yang X Gan J Chen S Xiao ZX Cao Y. CB-Dock2: improved protein-ligand blind docking by integrating cavity detection, docking and homologous template fitting. Nucleic Acids Res. (2022) 50:W159–w164. doi: 10.1093/nar/gkac394, PMID: 35609983 PMC9252749

[35] Morris GM Huey R Olson AJ. Using AutoDock for ligand-receptor docking. Curr Protoc Bioinformatics. (2008) 8:14. doi: 10.1002/0471250953.bi0814s24, PMID: 19085980

[36] Ren S Sun C Zhai W Wei W Liu J. Gaining new insights into the etiology of ulcerative colitis through a cross-tissue transcriptome-wide association study. Front Genet. (2024) 15:1425370. doi: 10.3389/fgene.2024.1425370, PMID: 39092429 PMC11291327

[37] Meleiro M Henrique R. Epigenetic alterations in glioblastoma multiforme as novel therapeutic targets: a scoping review. Int J Mol Sci. (2025) 26:634. doi: 10.3390/ijms26125634, PMID: 40565099 PMC12192589

[38] Madeshwaran A Vijayalakshmi P Umapathy VR Shanmugam R Selvaraj C. Unlocking estrogen receptor: structural insights into agonists and antagonists for glioblastoma therapy. Adv Protein Chem Struct Biol. (2024) 142:1–24. doi: 10.1016/bs.apcsb.2024.06.001, PMID: 39059983

[39] Lee E Sidoryk-Wegrzynowicz M Yin Z Webb A Son DS Aschner M. Transforming growth factor-α mediates estrogen-induced upregulation of glutamate transporter GLT-1 in rat primary astrocytes. Glia. (2012) 60:1024–36. doi: 10.1002/glia.22329, PMID: 22488924 PMC3353324

[40] Seshacharyulu P Ponnusamy MP Haridas D Jain M Ganti AK Batra SK. Targeting the EGFR signaling pathway in cancer therapy. Expert Opin Ther Targets. (2012) 16:15–31. doi: 10.1517/14728222.2011.648617, PMID: 22239438 PMC3291787

[41] Poteet E Liu D Liang Z Van Buren G Chen C Yao Q. Mesothelin and TGF-α predict pancreatic cancer cell sensitivity to EGFR inhibitors and effective combination treatment with trametinib. PLoS One. (2019) 14:e0213294. doi: 10.1371/journal.pone.0213294, PMID: 30921351 PMC6438513

[42] Vredenburgh JJ Desjardins A Reardon DA Friedman HS. Experience with irinotecan for the treatment of malignant glioma. Neuro-Oncology. (2009) 11:80–91. doi: 10.1215/15228517-2008-075, PMID: 18784279 PMC2718962

[43] Petitprez A Larsen AK. Irinotecan resistance is accompanied by upregulation of EGFR and Src signaling in human cancer models. Curr Pharm Des. (2013) 19:958–64. doi: 10.2174/1381612811306050958, PMID: 22973964

[44] Borgundvaag B Kudlow JE Mueller SG George SR. Dopamine receptor activation inhibits estrogen-stimulated transforming growth factor-alpha gene expression and growth in anterior pituitary, but not in uterus. Endocrinology. (1992) 130:3453–8. doi: 10.1210/endo.130.6.1534540, PMID: 1534540

