# Supplementary figures and images for: Integrative multi-omics analysis identifies TGFA as a novel glioma susceptibility gene and therapeutic target
Source: Front Neurol. 2025 Nov 5;16:1656490. doi: 10.3389/fneur.2025.1656490 (PMC12626838; doi:10.3389/fneur.2025.1656490)

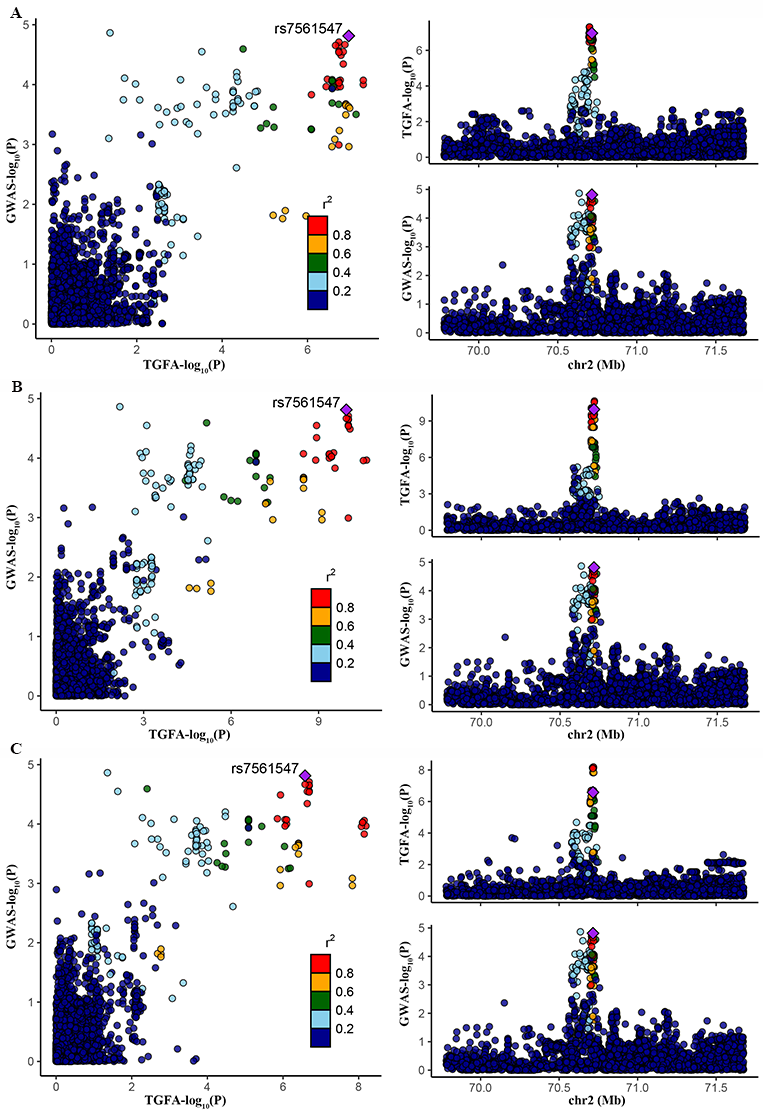

Supplement: Supplementary file 1 [file Data_Sheet_1.zip › supplemental material/Figure S/Figure S1 Regional association plot for colocalization analysis of TGFA protein with glioma risk.tif]

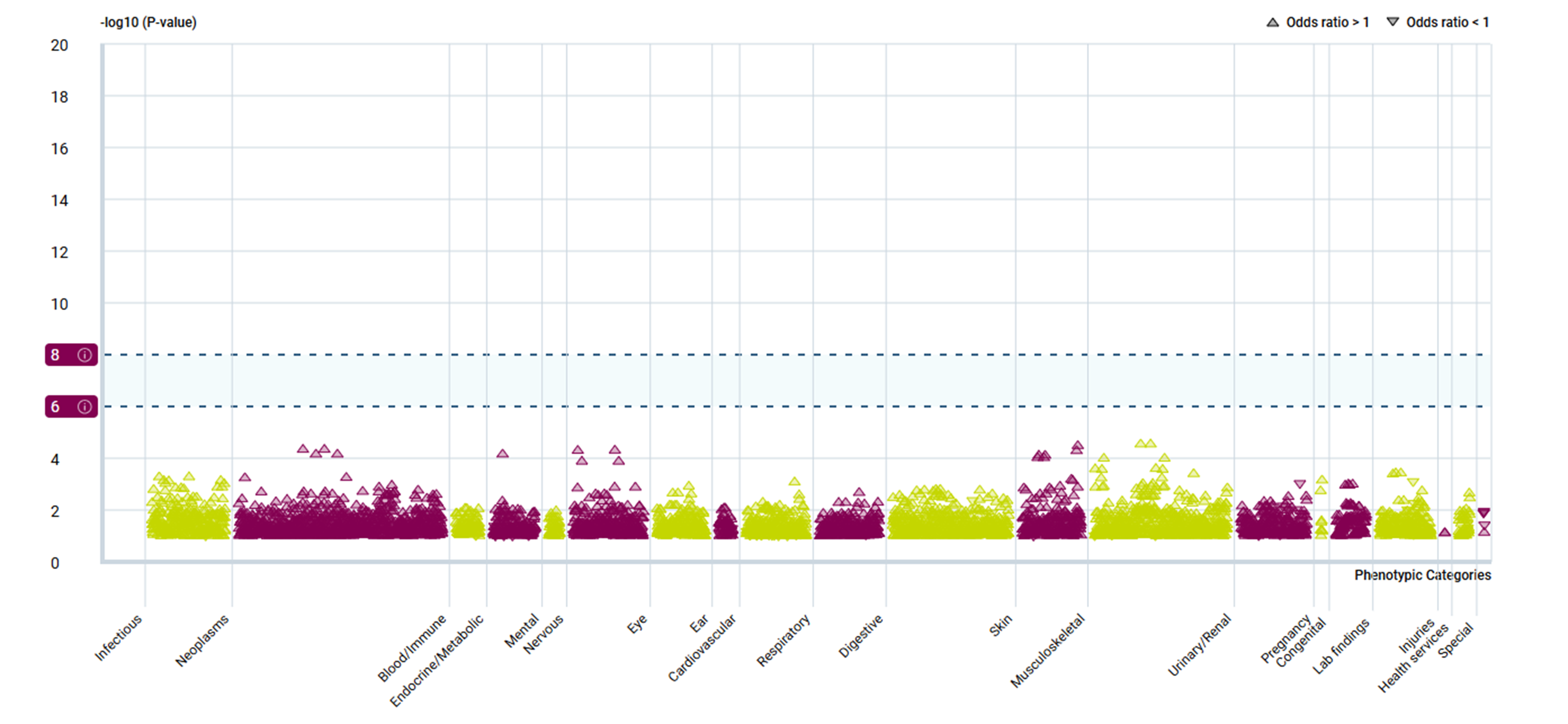

Supplement: Supplementary file 1 [file Data_Sheet_1.zip › supplemental material/Figure S/Figure S2 Phewas.tif]

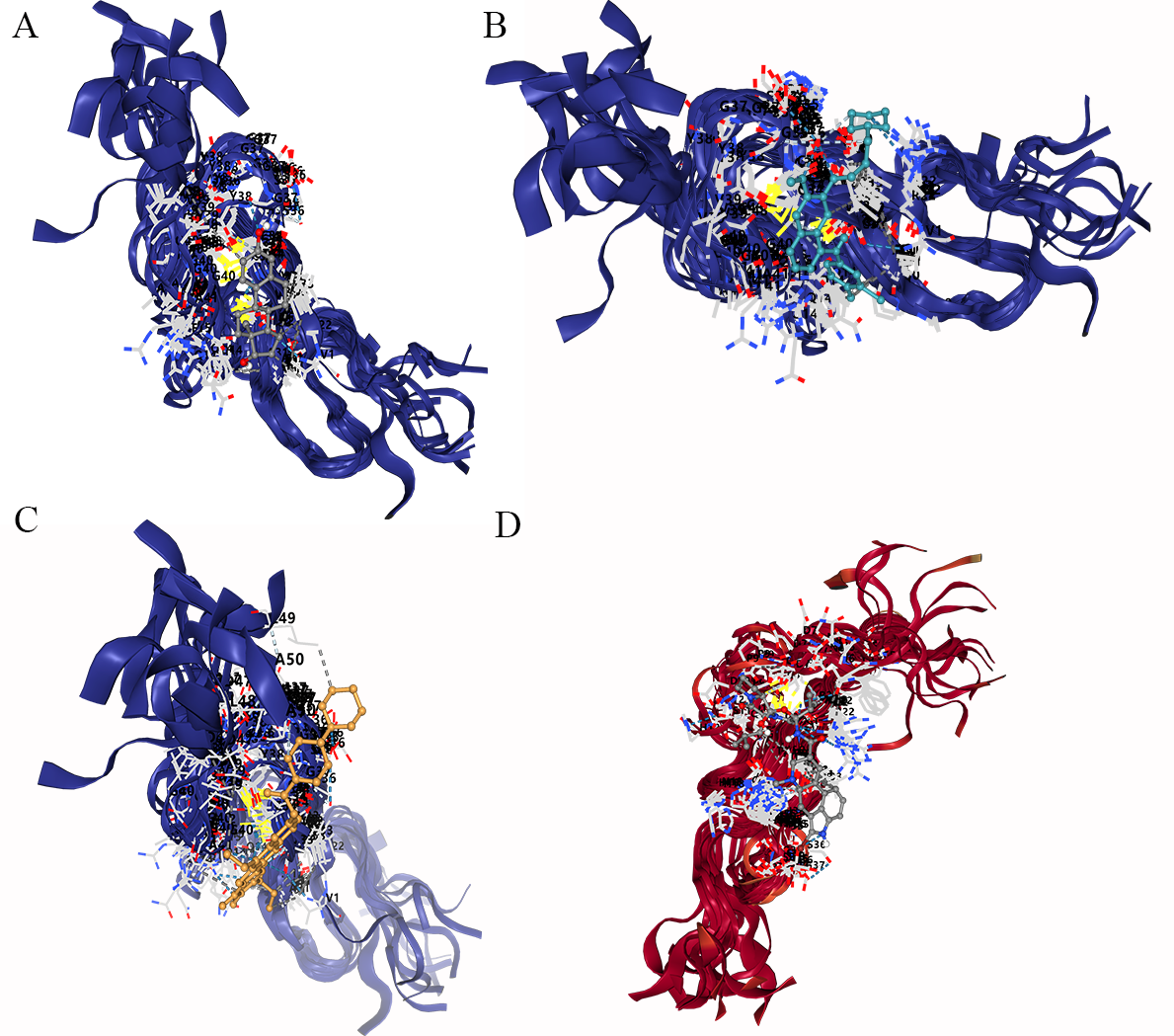

Supplement: Supplementary file 1 [file Data_Sheet_1.zip › supplemental material/Figure S/Figure S3 the molecular docking.tiff]

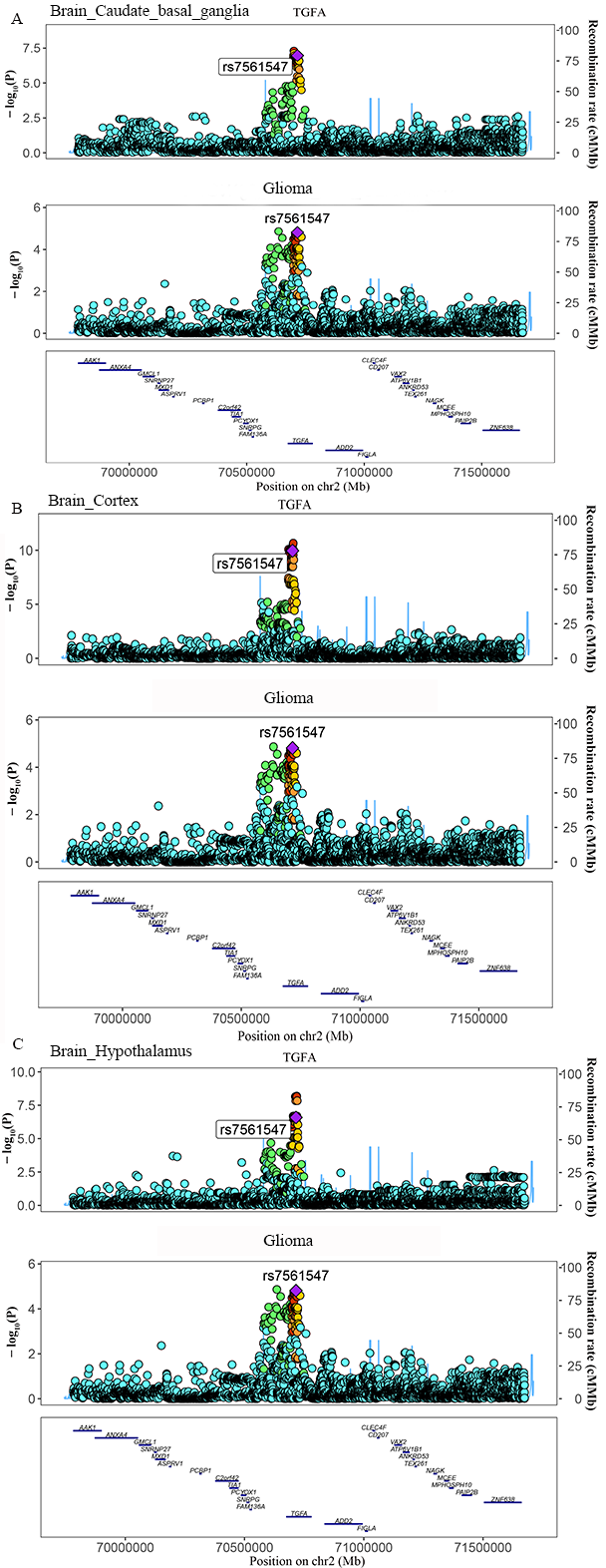

Supplement: Supplementary file 3 [file Image_2.tif]
